# Design and Development of an Integrated Virtual Reality (VR)-Based Training System for Difficult Airway Management

**DOI:** 10.1109/JTEHM.2025.3529748

**Published:** 2025-01-14

**Authors:** Saurabh Jain, Bijoy Dripta Barua Chowdhury, Jarrod M. Mosier, Vignesh Subbian, Kate Hughes, Young-Jun Son

**Affiliations:** Department of Industrial Engineering and Operations ResearchIndian Institute of Technology Bombay 29491 Mumbai 400076 India; Department of Systems and Industrial EngineeringThe University of Arizona 8041 Tucson AZ 85721 USA; Department of MedicineThe University of Arizona 8041 Tucson AZ 85721 USA; Department of Emergency MedicineThe University of Arizona 8041 Tucson AZ 85721 USA; Department of Biomedical EngineeringThe University of Arizona 8041 Tucson AZ 85721 USA; Edwardson School of Industrial EngineeringPurdue University 311308 West Lafayette IN 47907 USA

**Keywords:** Emergency procedure, endotracheal intubation, medical simulation, sensory system, virtual reality

## Abstract

For over 40 years, airway management simulation has been a cornerstone of medical training, aiming to reduce procedural risks for critically ill patients. However, existing simulation technologies often lack the versatility and realism needed to replicate the cognitive and physical challenges of complex airway management scenarios. We developed a novel Virtual Reality (VR)-based simulation system designed to enhance immersive airway management training and research. This system integrates physical and virtual environments with an external sensory framework to capture high-fidelity data on user performance. Advanced calibration techniques ensure precise positional tracking and realistic physics-based interactions, providing a cohesive mixed-reality experience. Validation studies conducted in a dedicated medical training center demonstrated the system’s effectiveness in replicating real-world conditions. Positional calibration accuracy was achieved within 0.1 cm, with parameter calibrations showing no significant discrepancies. Validation using Pre- and post-simulation surveys indicated positive feedback on training aspects, perceived usefulness, and ease of use. These results suggest that the system offers a significant improvement in procedural and cognitive training for high-stakes medical environments.

Clinical Impact: The developed VR-based simulation system significantly enhances airway management training by providing access to objective data on operator performance and decision-making under complex scenarios. This data allows for targeted feedback and tailored educational strategies improving clinician competency. Additionally the system’s potential for extension into mixed reality creates exciting possibilities for procedural guidance. By overlaying simulation cues onto the physical environment it can support real-time clinical decision-making and enhance situational awareness. This integration not only enriches the training experience but also prepares healthcare professionals for the complexities of real-world scenarios. Ultimately these advancements contribute to improved patient safety and outcomes in critical care settings.

## I. Introduction

The traditional “see one, do one, teach one” model for teaching medical procedures is potentially harmful to patients, especially for high-risk procedures, and is obsolete in modern medicine. As a result, high-fidelity simulation has risen in an effort to provide realistic alternative teaching modalities for these procedures in a safe environment. This simulation-based approach can provide experience and practice with high-risk or infrequent procedures through repetitive and deliberate practice.

Tracheal intubation is the quintessential procedure to utilize simulation. It is a complex and high-risk, yet commonly performed procedure in critically ill patients. Complication rates with intubation are reported as high as 50%, with cardiac arrest occurring between 2-4% of intubations. Risk increases dramatically when more than one attempt is required. Such threats to patient safety have made medical simulation a necessary tool to reduce this risk [1]. The management of difficult airways presents a significant challenge in the clinical practice, both for physicians and nursing staff, who play a critical role in airway management. The unpredictable nature of difficult airway scenarios requires not only procedural but also decision-making frameworks to avoid errors that compromise the patient safety. Studies report judgement errors such as to anticipate airway difficulty or persistence with failing strategies as common contributors to adverse outcomes in difficult airway scenarios [2]. These errors, compounded by stress induced from concurrent physiological deterioration, further emphasize the need for effective training. From a nursing perspective, the role extends beyond assisting in procedures to preparing equipment, monitoring patient vitals, and acting as a secondary line of defense during emergencies. Training programs for nurses are often underemphasized in traditional medical education frameworks, even though their contributions are crucial to the success of difficult airway management. Addressing these gaps is vital to advancing patient safety in critical care settings.

While current high-fidelity simulation capabilities provide anatomically reasonable representations of airway anatomy for procedural dexterity, they are limited in their ability to provide immersive and dynamic scenarios that induce stress in learners to reinforce or study decision-making. Further, newer devices such as video laryngoscopes that overcome anatomic obstacles to intubation expose the limitations of simulation for inducing stress to train cognitive performance [3].

Judgement errors during difficult airway management are common, even in experienced clinicians, and lead to patient harm [4]. Failure to identify and prepare for potential difficulty, and persistence with a strategy in the face of failure are common judgement errors, which are compounded by stress induced from concurrent physiologic deterioration. Existing simulation methods do not offer the ability to realistically represent the complex, dynamic scenarios needed to inject cognitive stress from either failure or physiological deterioration. These limitations diminish the ability to conduct research on, or train for, cognitive tasks, stress inoculation, or improve patient safety with simulation in modern airway management.

We designed and developed a Virtual Reality (VR)-based simulator for airway management to address these limitations by combining anatomic 3D-printed physical models and their virtual counterparts in a virtual environment. The system is designed to realistically mimic clinical settings. One-to-one mapping synchronizes the virtual representation of physical and virtual objects, and physical manipulation of the models during the procedure. Real-time interactions between the laryngoscope or endotracheal tube and the anatomical structures facilitate haptic feedback to the user in the physical environment. These interactions in physical space inform soft-tissue deformation in the virtual space through real-time analysis of the pressure profile along the laryngoscope to assign appropriate parameters to the anatomical structures. Finally, a multi-modal sensing framework is incorporated into the simulation and the physical environment to collect high-fidelity data in both environments. These features allow operators to experience comprehensive real-world intubation simulations with a broad spectrum of stress-inducing triggers.

This paper will review the current literature (Section II), describe the system development and implementation (Section III), report calibration and validation of the system (Section IV), and describe future development plans (Section V).

## II. Literature Review

Tracheal intubation in critically ill patients is a high-stakes procedure with significant risks. Nearly half of critically ill patients experience life-threatening complications related to intubation [5], [6], and the incidence of cardiac arrest during or shortly after the procedure is alarmingly high, affecting one in 25 to 30 patients. This risk is further amplified in patients with hypoxemic respiratory failure or hypotension [7]. Historically, the success and safety of intubation were predominantly associated with the clinician’s skill in direct laryngoscopy (DL), which involves overcoming anatomical barriers to align the airway’s visual axes for successful tracheal tube placement. Tongue pressure exerted by the laryngoscope has been objectively used to evaluate performance, while force measurements serve as a benchmark for comparing laryngoscope designs [8], [9]. Anatomically “difficult airways” requires more than two laryngoscopy attempts or taking over 10 minutes to intubate—are reported in 11–50% of cases outside the operating room (OR) [10], [11]. Unfortunately, methods for predicting difficult airways remain only modestly reliable [8], [9], [10], [11], and recent literature reveals that risk accumulates significantly even during first attempts, rather than during subsequent attempts [1].

The challenges of airway management extend beyond anatomical difficulty to encompass physiological derangements, such as those caused by induction of anesthesia, apnea, or positive pressure ventilation. These factors, often termed the “physiologically difficult airway,” introduce the risk of decompensation even when first-attempt laryngoscopy is successful [12], [13]. This complexity underscores that risk is not solely a function of technical skill with the laryngoscope but rather a product of intricate interactions between anatomical, physiological, and environmental factors [14].

Current simulation technologies, such as high-fidelity simulators, have attempted to address some of these challenges. These tools offer adjustable anatomical scenarios, including tongue swelling, pharyngeal edema, laryngospasm, and cervical immobility, providing a controlled environment for developing procedural skills. While they enhance direct laryngoscopy training and skill acquisition, their ability to replicate dynamic physiological and cognitive challenges remains limited [6], [8], [14]. Additionally, advances in devices like video laryngoscopes continue to highlight gaps in understanding and predicting anatomical difficulties and managing stress induced by physiological derangements. Clinical teaching through OR-based intubation further exacerbates these gaps, as difficult intubations are rare in the OR, occurring in only 1–2% of cases [8], [15], [16]. These factors collectively emphasize the need for comprehensive simulation approaches capable of addressing both procedural and cognitive challenges in airway management.

Virtual reality (VR) represents a transformative innovation in medical education, offering immersive, repeatable, and cost-effective training environments. Unlike traditional simulators, VR-based systems allow for the simulation of complex, high-stakes scenarios that are challenging to replicate in clinical settings. For example, Casso et al. and Samosorn et al. demonstrated VR’s versatility in enhancing skills across diverse medical procedures, from ophthalmologic examinations to arthroscopy [17], [18]. Moreover, VR provides a learner-centric approach that emphasizes both procedural competency and emotional engagement. Studies such as Moll-Khosrawi et al. underscore VR’s ability to foster empathy by bridging cognitive and emotional gaps between healthcare providers and patients [19].

Technological advancements in VR, particularly in haptic feedback, have significantly enhanced its potential for medical training. Kapoor et al. highlighted the importance of tactile engagement in simulating surgical procedures, while AR (augmented reality) applications explored by Shin et al. and Richards et al. demonstrated how immersive technologies can blend theoretical knowledge with hands-on applications [20], [21], [22]. These innovations allow for more nuanced skill development by incorporating realistic feedback mechanisms and interactive experiences.

Additionally, VR is redefining the retention and application of complex knowledge in medical education. Studies by Aebersold et al. and Weller et al. revealed that VR not only improves knowledge retention but also engages learners more effectively than traditional methods [23], [24]. In scenarios requiring high cognitive loads, VR simulations provide valuable insights into trainee decision-making processes, as noted by Ruiz-Rabelo et al., through metrics like eye movement and biometric parameters [25], [26], [27]. Such data-driven approaches offer unique opportunities for personalized and performance-based training.

The adaptability of VR and AR has been particularly evident during disruptions such as the COVID-19 pandemic. Malik et al. demonstrated how immersive technologies sustained educational continuity, accommodating diverse learning preferences while maintaining outcomes [24], [28]. Medical trainees utilizing VR simulation were 275% more confident, four times more focused, 400% faster in acquiring skills, and approximately 400% more emotionally connected to the content than classroom learners in one study simulating operating room fires [31]. In addition to training applications, VR provides a viable modality to study the difference in decision-making skills between varying levels of expertise. Ramos et al. [32], suggested that 8.3% of experts made incorrect decisions compared to 17.2% of novices in Advanced Cardiac Life Support simulations. These advancements underline the pivotal role VR plays in modernizing medical education and ensuring resilience in training frameworks.

Despite these advances, challenges persist. Current VR systems often lack realistic haptic feedback, limiting their effectiveness in training for complex procedures like tracheal intubation. However, hybrid approaches that combine VR with physical simulation technologies show promise in addressing these limitations. By integrating immersive virtual environments with tangible, hands-on components, such systems can provide comprehensive training that bridges the gaps in existing educational methods.

To summarize the current state of simulation technologies and highlight the advancements proposed in this study, Table 1 compares the gaps in existing simulators with the features of the proposed hybrid VR-based simulator:

**TABLE 1:** Gaps in existing simulation technologies vs. features of the proposed system.

Simulator	Highlighted Gaps	Proposed Simulator
High-Fidelity Manikins	Limited realism in emotional responses and complex scenarios [39]	Provides immersive environments with customizable emotional and clinical challenges.
Task Trainers	Lack of comprehensive clinical context [1]	Enhances context by integrating procedural and cognitive elements in realistic airway management scenarios.
Standardized Patients	Variability in performance and feedback quality [40]	Delivers consistent, data-driven feedback based on high-fidelity physical and cognitive metrics.
Low-Fidelity Simulators	Limited skill transfer to high-stakes environments [41]	Offers advanced calibration techniques ensuring precise tracking and realistic physics-based interactions.
VR/AR Simulators	Technical limitations and accessibility issues [42]	Combines tangible interfaces with immersive VR to create an accessible, hybrid simulation system.

The proposed system addresses these challenges through advanced features that integrate immersive virtual environments with realistic haptic and cognitive feedback mechanisms. Thus, it facilitates a scalable and effective solution for high-stakes training in tracheal intubation, bridging the procedural, cognitive, and emotional gaps identified in existing systems.

## III. System Architecture

**FIGURE 1.**
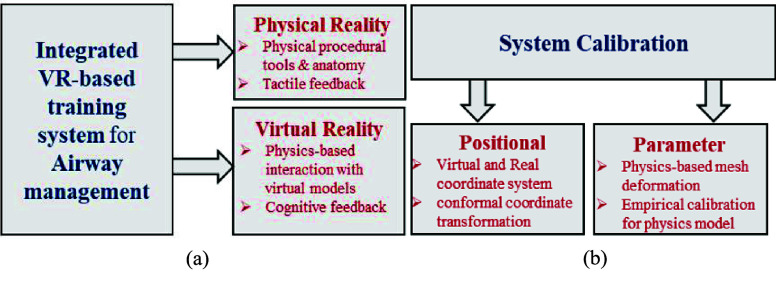
Conceptual Diagram for (a) system design (revised from Jain et al., 2020) and (b) system calibration.

An effective training system requires: (1) a suitable mode of training, (2) clear objectives, (3) translation of the objectives into system requirements, (4) implementation of those requirements, and (5) verification and validation of the system [29]. We designed and developed our VR-based system based on these requirements with a transdisciplinary team of emergency and critical care medicine physicians, simulation experts, and systems and biomedical engineers. End-users (e.g., medical students, residents, educators, and researchers) were engaged in an iterative prototype design process to develop and test the system. The resulting system is outlined in Fig. 1, which combines the physical and virtual environments [30], [31].

The proposed system consists of two interconnected components: the physical environment and the virtual environment. The physical environment includes tangible elements such as a manikin and procedural tools (e.g., laryngoscope and endotracheal tube) that are tracked in real time. The virtual environment, on the other hand, simulates an emergency room setting, incorporating virtual representations of the manikin and procedural tools to create a cohesive and immersive experience. The physical environment delivers haptic feedback through real-time physical interactions between the laryngoscope, endotracheal tube, and the manikin. Complementing this, the virtual environment provides visual and cognitive feedback to the user by overlaying real-time mesh deformation of anatomical soft tissues and simulating a photo-realistic clinical setting. The system workflow comprised of three phases:

1) **Preparation Phase:** The user begins by wearing the VR headset, which provides an immersive view of the virtual emergency room. Calibration ensures that the physical tools are tracked and aligned with their virtual counterparts. The system presents an introductory navigation to familiarize the user with the task objectives and interface through audio guidance.
2) **Simulation Phase:** During the session, the user physically interacts with the manikin and procedural tools, performing endotracheal intubation. These interactions are tracked in real time, with haptic feedback provided through the physical tools. Simultaneously, the virtual environment overlays visual feedback, including real-time mesh deformation of anatomical structures. Dynamic scenarios, such as vital sign changes, are incorporated to simulate stress and enhance decision-making skills.
3) **Self-Assessment Phase:** The immersive nature of the system allows users to immediately identify successes and challenges during the procedure. Key cues, such as visual changes in the virtual environment or the physical responses of the manikin, prompt users to self-assess their actions. The system reinforces correct techniques through real-time visual feedback, such as corresponding anatomical deformations based in their action during intubation, while highlighting lack of task completion. These features enable learners to iteratively refine their skills across multiple sessions.

Integrating the two environments with realistic real-time synchronization requires realistic physics-based interactions along with accurate visual representations using precise parametrization of the physics model. To accomplish this, we conducted system calibration through two approaches: positional and physics parameter calibration. Positional calibration involved establishing a one-to-one mapping between virtual and physical coordinate systems using conformal coordinate transformation to ensure accurate alignment. For physics parameter calibration, an empirical method was employed to determine parameters for soft-tissue movement, given that ground truth data for these parameters were unavailable. Together, this comprehensive calibration process resulted in the system’s capability to deliver haptic feedback and realistic cognitive feedback to users.

The user executes the procedure both physically and virtually. While the physical interaction ensures accurate haptic feedback, the virtual environment immerses the user in a realistic emergency room setting, simulating visual and cognitive challenges faced in real-world scenarios.

**FIGURE 2.**
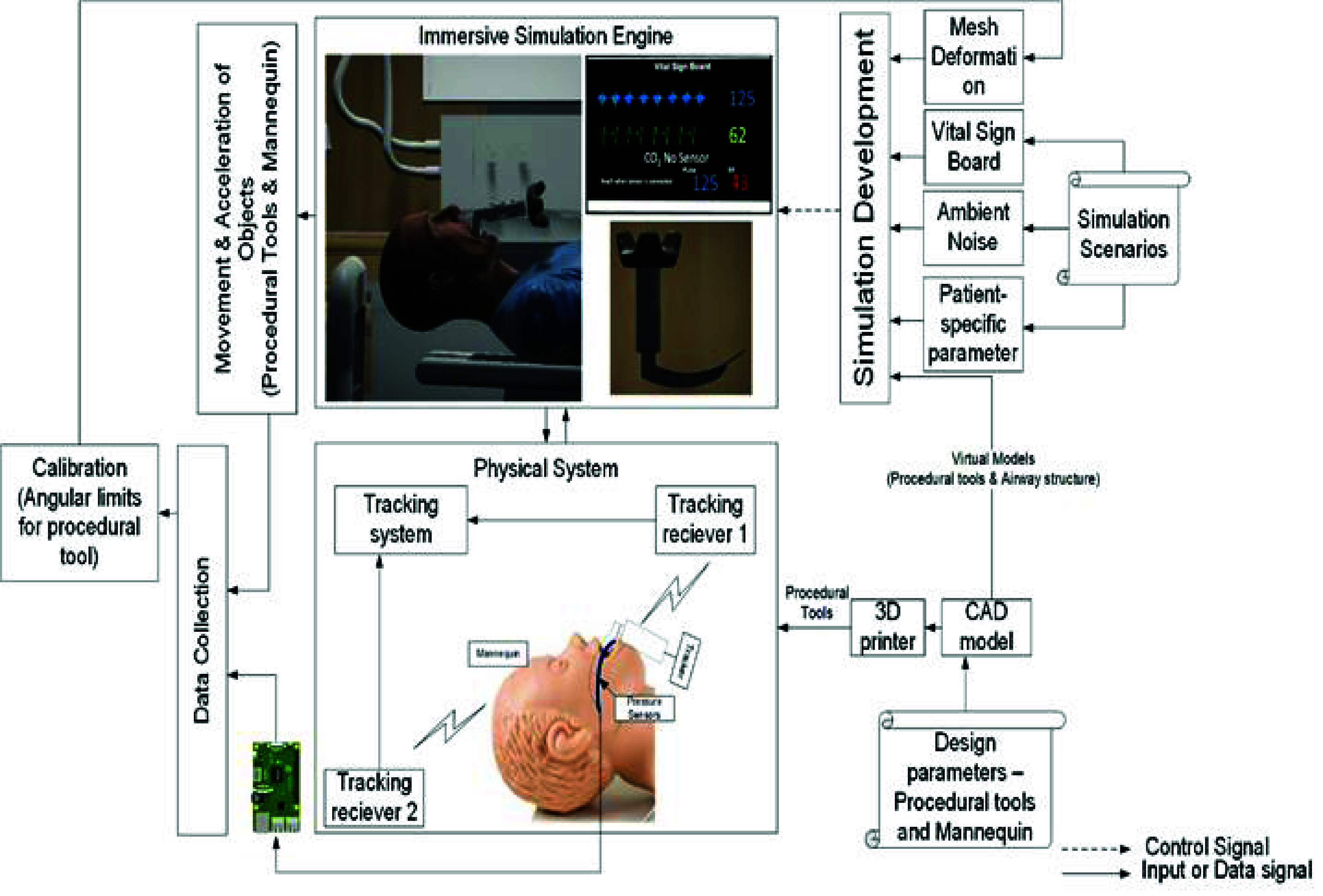
Overall framework of Integrated VR-based training system for airway management.

Our system was developed in three major phases: (1) simulation configuration, (2) simulation execution, and (3) dynamic data collection. Simulation configuration included designing and developing 3D models to precisely replicate both the manikin’s anatomical structures and the intubation equipment (laryngoscope, endotracheal tube, stylet). These 3D models were scaled one-to-one in regards to their physical counterparts and subsequently imported into the Unity 3D simulation game engine. The physical components (laryngoscope and stylet) were modelled and produced using computer-aided design and additive manufacturing processes, respectively. Pressure and position sensing modules were placed on the laryngoscope and stylet to stream real-time data informing virtual soft-tissue deformation and device position based on the performance of the user. The user interacts with the physical models while viewing them through the VR room-scale headset (HTC Vive Pro, HTC, Taiwan). Back-end data collection from sensors allows offline analyses of the technical and cognitive performance of the user. The system design (Fig. 2) was then refined through system implementation and data-driven simulation.

### A. System Design

We followed participatory [32], [33] and V-model approaches to systems engineering [32] to identify the appropriate mode of delivery for intubation. The V-model represents various stages in the software development life cycle where system requirements are established in collaboration with end-users, then outlined in a specification document of technical components and data flow, and then segregated into specific component modules—in this case laryngoscopy, vital sign monitoring, and endotracheal tube insertion. We then derived a low-level module specification for each component, which comprised of functionalities and interfaces between each of the components to facilitate high-fidelity user interaction. Once the system was developed and implemented, we performed the testing process in reverse order to eliminate potential issues starting from lower-level modules. The testing phase was followed by integration testing and system testing for system performance. Lastly, we conducted acceptance testing to confirm successful implementation of the system using validation studies with clinical domain experts.

The laryngoscopy requirements that were established by clinical experts where video laryngoscopy (VL) as the laryngoscope and physiologic deterioration during the simulations. Table 2 shows the complete set of basic requirements as determined by the clinicians.

**TABLE 2:** System requirements.

Design components	Requirements
Patient environment	1. Rendering a photorealistic patient environment 2. Ability to simulate different scenarios using visual, auditory, and haptic feedback 3. Ability to render live video feed from the virtual camera on laryngoscope 4. Flexibility to test patient environment configurations (e.g., intensive care unit, emergency department, operating room, prehospital, battlefield)
Procedural tools	1. Laryngoscope (a) Ability to track physical movements in the virtual environment (b) Ability to realistically represent laryngoscope-soft tissue interactions (c) Virtual camera and light source within immersive environment 2.Endotracheal tube (a) Ability to track physical movements in the virtual environment
Anatomical structures	1. Well-segmented airway structures to provide realistic anatomy 2. Accurate placement of critical structures and abnormalities 3. Accurate calibration of virtual anatomical structures with physical manikin
Back-end data collection module	1. Limited physical-virtual reality lag time (i.e., Ability to stream real-time force pressure distribution along the laryngoscope into the simulation) 2. Real-time analysis of data (e.g., pressure distribution and location information of procedural tools) from multi-modal sensors

**FIGURE 3.**
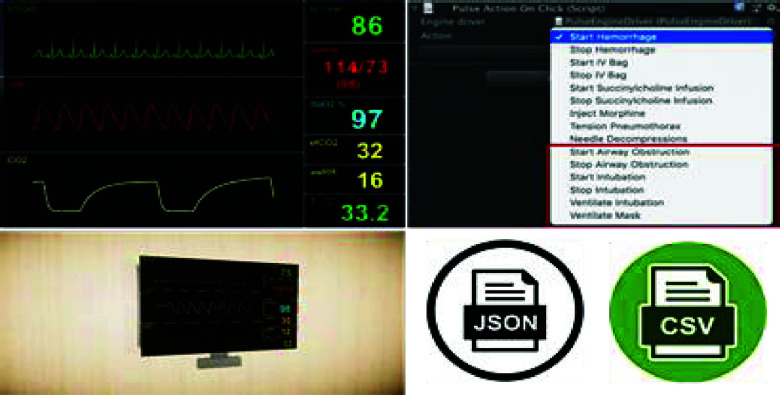
Vital sign monitor using Pulse physiology engine providing the capability to assign the input physiological scenarios using .JSON or .CSV format.

**FIGURE 4.**
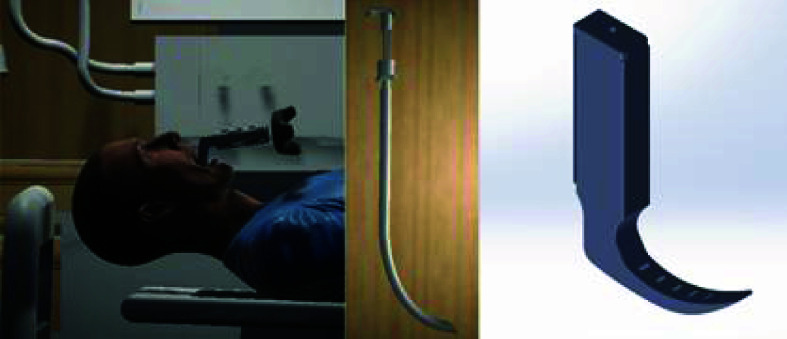
CAD models of the Anatomical structures (left), intubation tube (middle), and modified procedural tools (right).

The design of a patient environment requires photorealistic representation of the relevant environment (e.g., Emergency Department, Intensive Care Unit, Operating Room, prehospital, battlefield). A hospital room includes important features such as a video monitor for rendering the live video feed from the VL and a vital sign monitor (Fig. 3). We used a pulse physiology engine, an open-source API, to provide the user with an accurate vital sign monitor representation during the simulation. Integration of the pulse physiology engine provides accurate and consistent patient physiology and allows for patient-specific modeling in virtual simulations. The vital sign monitor provides the operator with dynamic patient conditions produced by this engine. Furthermore, the developed simulation model facilitated the execution of pre-defined simulation scenarios for different physiological conditions using JSON or CSV formats. Hence, it provides the flexibility to design and execute a wide range of instruction-driven simulations within a realistic environment. For the patient model and procedural tools, we developed 3D models using Computer Assisted Design (CAD). The virtual models were imported into the simulation environment (Unity 3D), and the physical models were 3D-printed using Fused Deposition Modeling (FDM). Our previous work [33], [34] employs forward and reverse engineering to construct CAD models based on the geometric information of the objects under consideration. SolidWorks 2021 [35] was used to develop the detailed CAD models, ensuring high precision and design accuracy. The physical prototypes were fabricated using the Creality Ender 3 [36] 3D printer, leveraging Fused Deposition Modeling (FDM) technology to create robust and accurate representations for testing and validation. Reverse engineering was utilized to model the anatomical structures based on the physical measurements from the actual manikin structures (Fig. 4). The physical procedural tools were modified to accommodate pressure and positioning sensors to facilitate data collection and empirical calibration. The laryngoscope prototype includes a mini-sensory circuit, providing real-time data pertaining to pressure profile along the blade during contact with the upper airway structures and transmits the data wirelessly to the Unity 3D game engine. We modeled the laryngoscope, endotracheal tube, and soft tissue positions using colliders and hinge joints (Fig. 4). Pressure values from the interaction between laryngoscope and tissue in the physical space inform appropriate physics parameters to represent dynamic tissue visualization and movement in the virtual space.

**FIGURE 5.**
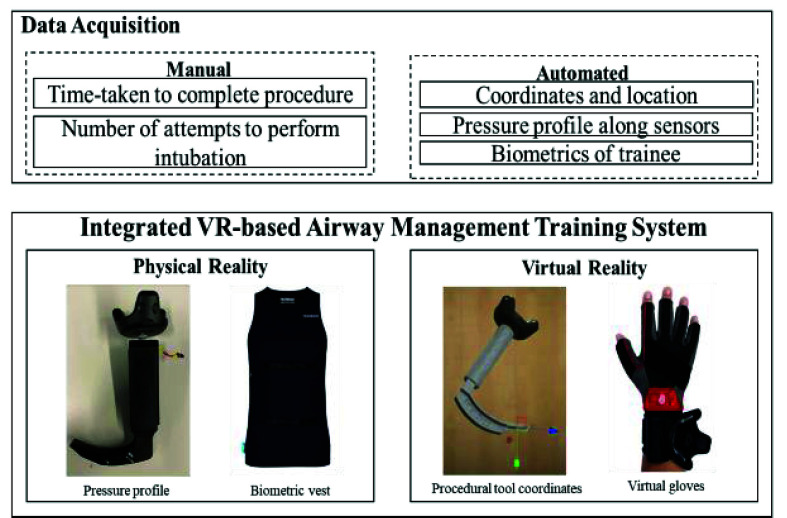
Data-acquisition using multi-modal sensory system.

The backend data collection module captures and utilizes real-time sensory data from: (a) HTC Vive VR headset, (b) pressure sensing module, and (c) Hexoskin biometric vest (Carré Technologies inc., Montréal, Canada) to drive the simulation (Fig. 5). The data pertaining to the pressure limits for the appropriate representation of the soft-tissue deformation based on the pressure entered by the user was incorporated. A data streaming frequency of 10Hz was used to ensure smooth data collection and real-time analysis without any lag. Background data are automatically collected from the procedural-related sensors, as well as user biometric parameters (heart rate variability, respiratory rate, etc.). Manual data are added such as high-level metrics (task-based metrics and completion times). The virtual patient room was tested for face validity of an accurate photorealistic representation. Additionally, the laryngoscope, endotracheal tube, and anatomical structures were validated for spatial dimensions within the virtual as well as physical environments to provide the user with a one-to-one mapped model.

We refined the modules to minimize any lag in the photo-realistic images within the VR environment and avoid motion sickness to the user (section IV-A.1), optimize real-time soft-tissue deformation (section IV-A.2), and validity and realism of the system (section IV-B).

### B. System Implementation

**FIGURE 6.**
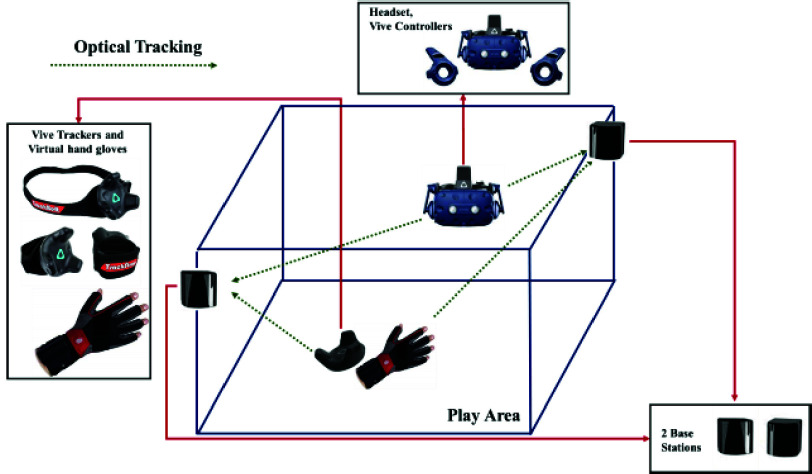
HTC Vive setup for virtual patient room.

A room-scale VR headset provides visual, auditory, and haptic feedback modeled within the Unity 3D simulation platform. The HTC Vive Pro headset provides a high-resolution head-mounted display (HMD) and a broad field of view along with room-scale tracking, and Unity 3D enables the physics-based movement of components within the virtual environment (Fig 6).

**FIGURE 7.**
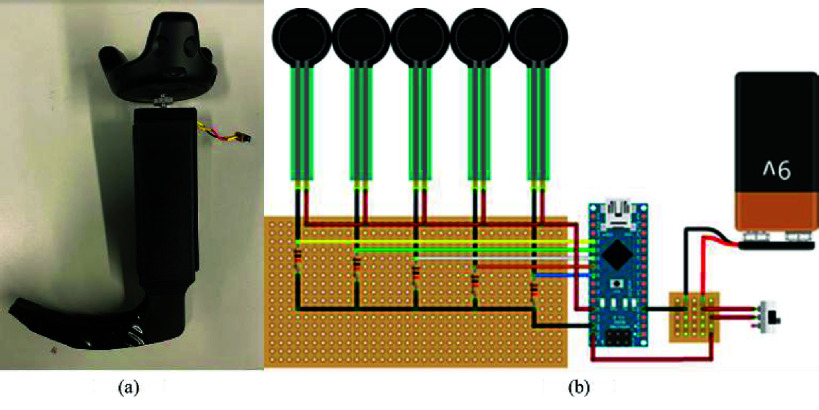
(a) Pressure sensing module within the modified laryngoscope, and (b) Circuit diagram for pressure sensing module. The system is comprised of five pressure sensors, Arduino nano BLE (Bluetooth Low Energy), battery, and resistors embedded in and on the laryngoscope.

Two auxiliary sensors track the modified laryngoscope and endotracheal tube, respectively, and a third sensor tracks the laryngoscopy ergonomics from the elbow position of the user. Gloves are worn to visualize the virtual hand placement and equipment manipulation. Sensory data are integrated using a unified framework (Fig. 7).

Positional calibration was performed by the 3D conformal coordinate transformation method. With 3D conformational coordinate transformation, the translation, rotation, and scaling matrices are obtained using the general form of 3D conformal transformation of virtual over the physical objects. The general form of 3D conformal coordinate transformation is shown below:

\begin{align*} \left [{{\begin{array}{cccccccccccccccccccc} X' \\ Y' \\ Z' \\ \end{array}}}\right ]= \lambda {\boldsymbol {R}} \left [{{\begin{array}{cccccccccccccccccccc} X \\ Y \\ Z \\ \end{array}}}\right ]+\left [{{\begin{array}{cccccccccccccccccccc} t_{x} \\ t_{y} \\ t_{z} \\ \end{array}}}\right ] \tag {1}\end{align*}

where $\lambda $ denotes the scale factor, $\boldsymbol {R}$ represents the rotation matrix and $t_{x}, ~t_{y}~and~t_{z}$ denotes the translation matrix about x, y, and z directions.

**FIGURE 8.**
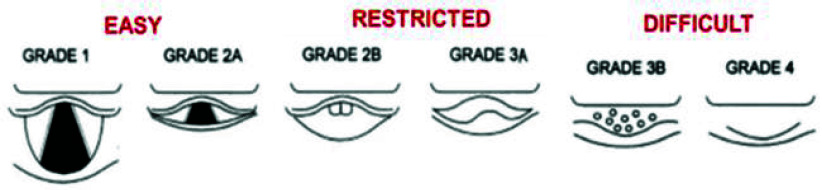
Easy, restricted, and difficult glottic views using Cormack-Lehane classification (revised from [1]).

Tracheal intubation requires laryngoscopy to align the visual airway axes [i.e., Pharyngeal (PA), Laryngeal (LA), and oral axes (OA) to adequately view the airway and place an endotracheal tube. Head and neck positioning bring the axes closer to parallel, and the laryngoscope compresses and displaces the tongue and epiglottis to view of the glottic opening, generally described using the Cormack-Lehane (C-L) grading system (Fig. 8.). Thus, a realistic VR-based simulation requires accurate modeling of compression and displacement of the upper airway structures. We calibrated the laryngoscopic pressures required to model this requirement (parameter calibration) by generating a distribution of pressure values using force sensing resistors on the laryngoscope (Fig 7) generated during multiple laryngoscopy repetitions at varying target C-L grades of view. Fig. 11 shows two configurations of the neck under the regular position scenario (left) and the scenario when the virtual patient is being operated utilizing the appropriate soft-tissue deformation based on the pressure distribution.

**FIGURE 9.**
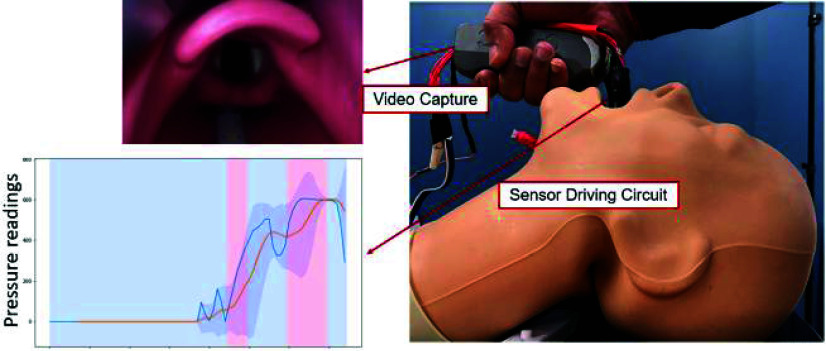
Experimental setup for deriving the pressure profile based for appropriate soft tissue deformation.

**FIGURE 10.**
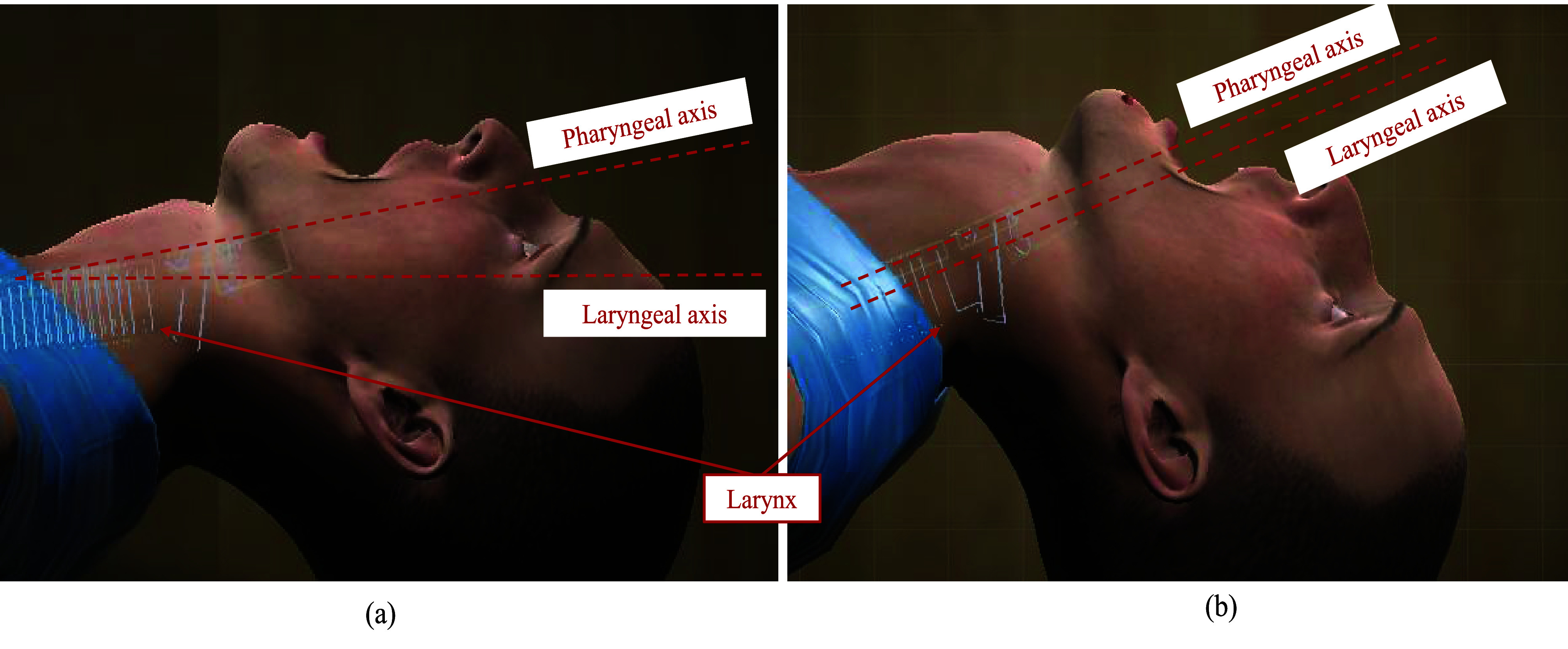
Calibration of soft-tissue deformation based on the Pharyngeal and Laryngeal axis. (a) The default configuration in the absence of any use of the procedural tool, whereas, when adequate pressure is applied using the procedural tool, (b) the alignment of the C-L view takes place.

**FIGURE 11.**
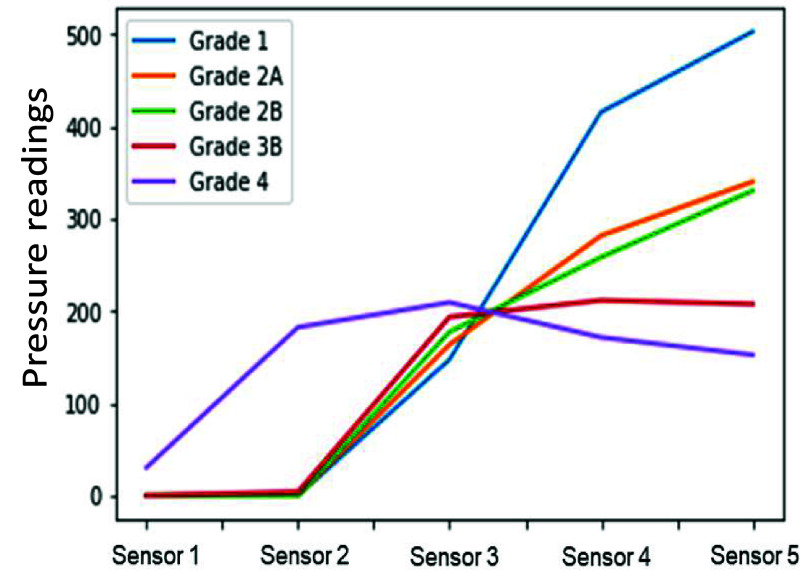
Mean pressure profiles along the CMAC laryngoscope for five different C-L views and deflated tongue configuration.

### C. Data-Driven Simulation

We calibrated the system for five different glottic views (C-L grades 1, 2A, 2B, 3B, and 4) (Fig. 8) using two different conditions.

First, five repetitions of each grade of view were obtained using our modified laryngoscope as a direct laryngoscope under routine conditions (tongue deflated). The tongue was then inflated with 50 ml of air, and the sequence was repeated. Then, the sequence was repeated using a modified commercially available identically shaped video laryngoscope (size 3 Karl Storz C-MAC video laryngoscope) with seven expert physicians to generate a distribution of pressure profiles for each grade of view (Table 3) across clinicians of varying experience, gender, clinical specialty, and airway difficulty (Fig 9). Live video feeds from the laryngoscope were recorded to calibrate the angular deformation for a particular glottic view against the corresponding pressure distribution.

**TABLE 3:** System configuration for data collection for parameter calibration.

Total number of subjects	Number of Replication for each scenario	C-L Views	Tongue status	Data
7	5	(a) 1 (b) 2A (c) 2B (d) 3B (e) 4	(a) Deflated (b) Inflated	(a) Time-stamped pressure profile (b) Video feed

**FIGURE 12.**
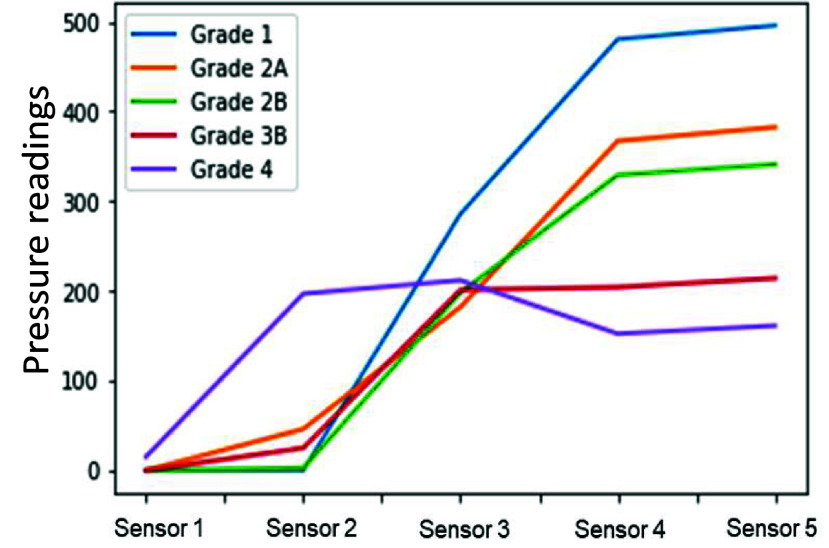
Mean pressure profiles along the CMAC laryngoscope for five different C-L views and inflated tongue configuration.

**FIGURE 13.**
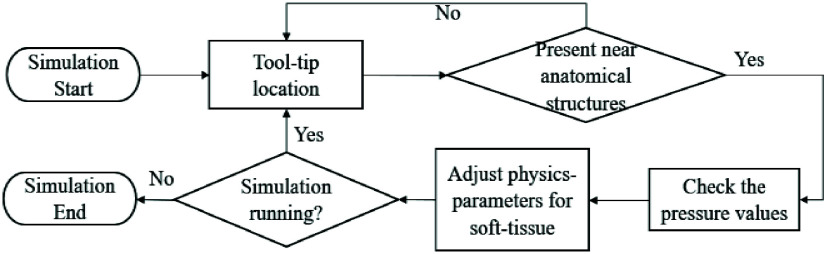
Flowchart for dynamic-data driven simulation execution of soft-tissue deformation based on position and pressure profile along the laryngoscope.

For this experiment, we defined an expert as a clinician with >100 intubations and >five years of post-training experience. The AirSim Advance X© manikin (trucorp, Lurgan, Ireland) was used for all experiments. It was also incorporated into our VR-based system to represent the physical environment. Fig. 12 and 13 show the mean pressure profiles obtained using the C-MAC video laryngoscope for deflated and inflated tongue configuration, respectively. These mean pressure profiles associated with each C-L grade view and tongue configurations allowed the approximation of pressure ranges for each configuration.

Based on the obtained pressure profiles, C# scripts were used to analyze the mean pressure values of each sensor within pre-defined ranges to identify the corresponding values of angular limits pertaining to soft-tissue deformation (Table 4). As the C-L views move from grade 1 to 4 (i.e., better to worse), there is a significant reduction in the pressure exerted on the tip of the laryngoscope blade (sensor 5) (Figs. 12 and 13). This trend suggests that the pressure exerted on the laryngoscope shifts towards the middle of the blade as the C-L view changes from grade 1 to 4 (i.e., tongue compression versus epiglottis elevation). Fig. 11 shows the implementation of the soft-tissue deformations based on the pharyngeal and laryngeal axes.

**TABLE 4:** Physics parameters based on pressure profile along procedural tool.

Configuration (Tongue situation – C-L grade)	Pressure sensors readings (Mean ± Standard Deviation)					Allowable angular deformation (degrees)
	Sensor 1	Sensor 2	Sensor 3	Sensor 4	Sensor 5	
Deflated – 1	0	0	$147.23~\pm ~11.82$	$415.34~\pm ~23.19$	$502.43~\pm ~22.97$	30.21
Deflated – 2A	0	0	$163.56~\pm ~8.69$	$281.45~\pm ~17.43$	$339.92~\pm ~18.52$	29.43
Deflated – 2B	0	0	$177.29~\pm ~13.57$	$258.12~\pm ~9.39$	$330.41~\pm ~21.79$	27.87
Deflated – 3B	0	$4.13~\pm ~0.43$	$193.43~\pm ~19.66$	$211.43~\pm ~11.23$	$207.32~\pm ~18.35$	21.23
Deflated – 4	$30.29~\pm ~6.34$	$182.21~\pm ~12.49$	$209.12~\pm ~24.31$	$171.31~\pm ~15.53$	$152.39~\pm ~20.01$	10.42
Inflated – 1	0	0	$285.87~\pm ~30.12$	$480.57~\pm ~26.45$	$495.87~\pm ~43.12$	37.77
Inflated – 2A	0	$46.12~\pm ~10.32$	$182.12~\pm ~21.43$	$367.21~\pm ~18.72$	$382.45~\pm ~28.71$	31.04
Inflated – 2B	0	$2.56~\pm ~1.23$	$198.24~\pm ~10.29$	$329.12~\pm ~17.97$	$341.21~\pm ~19.32$	29.89
Inflated – 3B	0	$24.98~\pm ~5.23$	$201.21~\pm ~19.21$	$204.28~\pm ~23.77$	$214.21~\pm ~27.91$	24.41
Inflated - 4	$15.24~\pm ~12.45$	$196.91~\pm ~11.89$	$211.89~\pm ~21.34$	$152.32~\pm ~14.81$	$161.29~\pm ~19.24$	14.53

**FIGURE 14.**
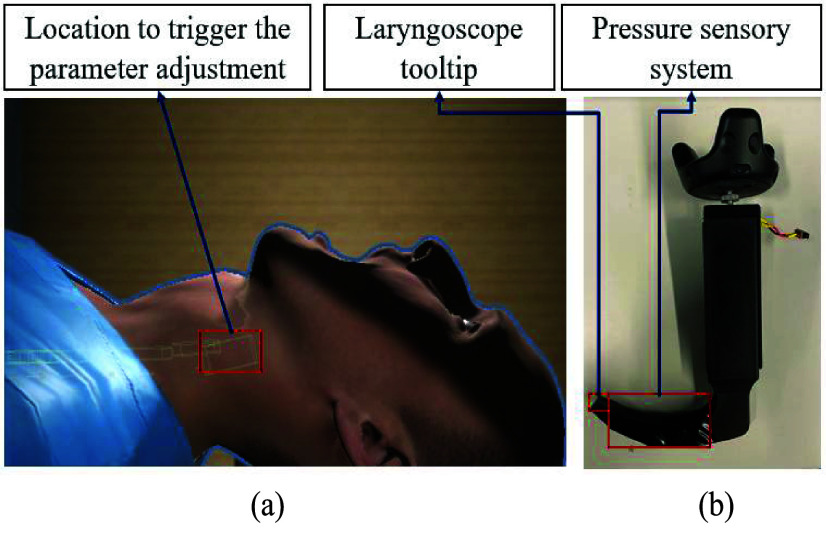
Key components for developed system, (a) location to trigger the soft-tissue deformation, and (b) Laryngoscope with tooltip and sensory system.

**FIGURE 15.**
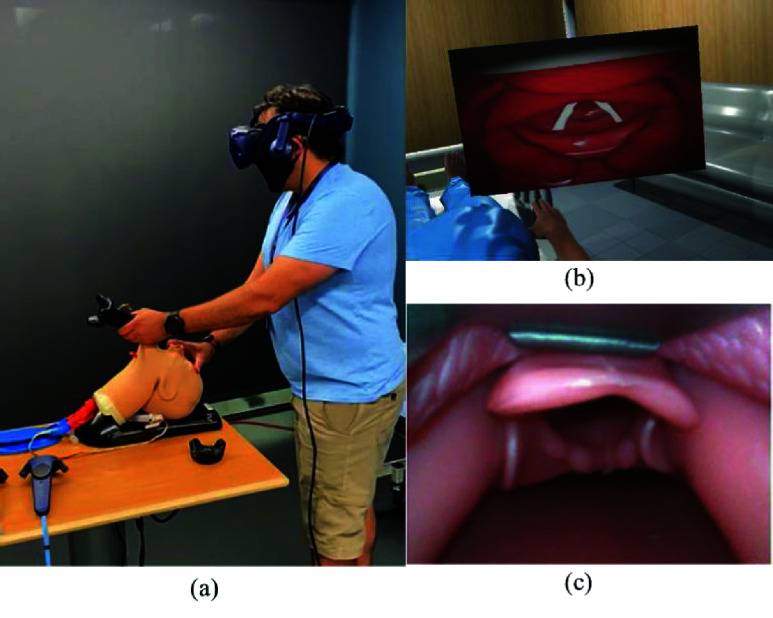
Experimental configuration for testing the parameter calibration. (a) Expert performing the procedure trial for grade 1 view utilizing the VR-based system, (b) Grade 1 view within the virtual world, and (c) Grade 1 view of the airway for comparative analysis utilizing the traditional systems.

Combined, the alignment of the laryngoscope at the trigger point (position) as well as the pressure profile allows the system to align the pharyngeal and laryngeal axes at the correct corresponding angle (Table 3). Both metrics are streamed in real-time and utilized by Unity 3D to simulate the continuous soft tissue movement (Fig. 14). We found that tracking the coordinate movement of the laryngoscope tool played an important role. Computational requirements (and thus lag), were reduced by streaming pressure values only when the laryngoscope tip approached the collider located near the goal anatomical structures (base of the tongue) (Fig. 15).

## IV. Validation Analyses

We objectively and subjectively conducted validation analyses. The objective validation evaluated the positional and parametric calibration accuracy, while the subjective validation evaluated technology acceptance by potential end-users [38].

### A. Calibration Experiments

#### A.1. Positional calibration

Several studies in image-guided therapy and medical simulation have established 0.1 cm errors as acceptable positional calibration [33], [34], which we adopted for our calibration experiments. Calibrated was validated using 10 subjects.

The experiment involved measuring the calibration error by placing the tip of the laryngoscope on a fixed anatomic landmark (entrance of the manikin mouth) in the physical reality. The Euclidean distance was calculated within the virtual environment between the actual and observed placement on the pre-defined anatomic landmarks. Each subject performed six trials to ensure robustness in terms of performance. The following hypothesis was established to test the mean calibration error (cp $_{\mathrm {error}}$) for boundedness within the suggested calibration error:

\begin{align*} {H_{0}\mathrm {:}cp}_{error}\mathrm {\le 0.1} \\ {H_{a}\mathrm {:}cp}_{error}\mathrm {> 0.1}\end{align*}

A t-test was conducted to evaluate the positional accuracy of as shown in Table 5, which suggests that the system ensures one-to-one positional mapping between the virtual and physical models with little variance.

**TABLE 5:** Overall T-Test Results for Positional calibration.

Total number of samples	Mean	Standard deviation	DoF	t-value	Pr > t
60	0.09591	0.03276	59	-0.96683	0.8312

#### A.2. Parametric calibration

Soft-tissue deformation was validated by extracting the physics parameters using the commercially available VL (CMAC) modified with the same five sensors and comparing with the simulation system using an expert clinician (Fig. 15). Laryngoscopes were performed with each system to a goal C-L grade of view, and pressure profiles were compared using a two-sample Hotelling T^2^ test. The pressure values along each configuration.

\begin{align*} H_{0}{:}\boldsymbol {\mu }_{1}{=}{\boldsymbol {\mu }}_{2}{} \\ H_{a}{:}{\boldsymbol {\mu }}_{1}{\ne }{\boldsymbol {\mu }}_{2}\end{align*}

where, $\begin{aligned} \boldsymbol {\mu }_{\mathbf {1}}\mathbf {=}\left ({{ \frac {\begin{array}{cccccccccccccccccccc} \mu _{11} \\ \mu _{12} \\ \boldsymbol {\vdots } \\ \end{array}}{\mu _{15}} }}\right) \end{aligned}$ and $\begin{aligned} \boldsymbol {\mu }_{\mathbf {2}}\mathbf {=}\left ({{ \frac {\begin{array}{cccccccccccccccccccc} \mu _{21} \\ \mu _{22} \\ \boldsymbol {\vdots } \\ \end{array}}{\mu _{25}} }}\right) \end{aligned}$ represent the mean pressure values along five sensors for CMAC $\left ({{ \boldsymbol {\mu }_{\mathbf {1}} }}\right)$ and VR $\left ({{ \boldsymbol {\mu }_{\mathbf {2}} }}\right)\mathbf {}$ system, respectively. The results of the two-sample hoteling t^2^ test are summarized in Table 6 for each configuration, suggesting similar pressure profiles between VR-based system and CMAC training system

**TABLE 6:** Overall results for Hotelling T^2^ test for different configurations.

Configuration (Tongue, C-L grade)	Number of active sensors	DOF	T^2^-value	P Value
Deflated, Grade 1	3	3, 66	0.5435	0.6541
Deflated, Grade 2A	3	3, 66	0.4860	0.6931
Deflated, Grade 2B	3	3, 66	0.3406	0.7959
Deflated, Grade 3B	4	4, 65	0.3965	0.8104
Deflated, Grade 4	5	5, 64	0.6353	0.6734
Inflated, Grade 1	4	4, 65	0.5417	0.7055
Inflated, Grade 2A	4	4, 65	1.1083	0.3602
Inflated, Grade 2B	4	4, 65	0.8213	0.5163
Inflated, Grade 3B	4	4, 65	0.8237	0.5148
Inflated, Grade 4	5	5, 64	0.8306	0.5326

### B. System Validation

Pre- and post-simulation surveys were conducted to evaluate the realism and functionality of the system, utilizing the Technology Acceptance Model (TAM) [38]. The TAM survey assessed factors such as realism, comparison with existing technology, and the feasibility of the VR-based training system on a 5-point Likert scale, with responses from 7 clinicians. These experiments were conducted at a Medical Education Center at the University, providing the platform for system validation under real-world training scenarios. Summary statistics for both the pre- and post-simulation surveys are provided in Tables 7 and 8, respectively.

**TABLE 7:** Post-simulation survey results showing the feasibility of VR-based for airway management procedure.

Feasibility and Realism		Mean score (Likert scale:1-5, N=7)	
Agreement with the following statement:		Simulation manikin	VR system
Feasibility of ETI with the utilized system	4.86	4.86	
Realism of the utilized system in representing an actual clinical intubation	4.43	4.43	
System Comparison		Mean score (Likert scale:1-3, N=7)	
Compared to the manikin/task trainer, I rate the virtual reality-based system as: inferior/similar/superior	2.57		
Perceived Usefulness of the VR system		Mean score (Likert scale:1-7, N=7)	
Using the system will improve my performance in airway management	5.71		
Using the system will enhance my effectiveness in airway management.	5.71		
I find the system to be useful in airway management	6		
Perceived ease of use of the VR system		Mean score (Likert scale:1-7, N=7)	
Interaction with the system is clear and understandable	5.71		
Interacting with the system does not require a lot of my mental effort	6		
System is easy to use	5.86		
Easiness on get system to do what I want it to do	5.43		

**TABLE 8:** Pre-Simulation survey results showing the demographic information and comfort level.

Total Experts	N=7 (%)
Age	
36-40	3 (42.85)
$\ge 41$	4 (57.15)
Gender	
Male	5 (71.42)
Female	2 (28.58)
Specialty	
Emergency Medicine	3 (42.85)
Critical care medicine	1 (14.30)
Pulmonary critical care medicine	3 (42.85)
Current role	
Attending Physician	7 (100.00)
Years of practice post training	
>5 years	7 (100.00)
Familiarity with Endotracheal intubation (ETI)	N=7 (%)
Comfort-level with ETI (1)–5 Likert)	
Somewhat comfortable	2 (28.57)
Extremely comfortable	5 (71.43)
Successful ETI under clinical setting	
76-100	1 (14.28)
>100	6 (85.72)
First-pass success rate for ETI under clinical setting	
61-75%	3 (42.85)
76-90%	2 (28.57)
>90%	2 (28.57)
Familiarity with simulation	N=7 (%)
Familiarity with ETI under simulation (1)–5 Likert)	
Slightly familiar	1 (14.58)
Moderately familiar	1 (14.58)
Extremely familiar	5 (71.42)
Successful ETI under simulation	
0-25	1 (14.28)
26-50	2 (28.57)
>100	4 (57.14)
Familiarity with VR	Mean score (Likert scale:1-5, N=7)
Comfort-level using VR	2.57
Knowledge/experiences using VR	1.71

The results demonstrated that users rated the system positively in terms of feasibility, perceived usefulness, and ease of use, particularly in relation to human-computer interaction and overall system behavior. This positive feedback indicates that the system performs well in a relevant, simulated clinical environment, positioning it for broader adoption in Airway Management training curriculums. Additionally, qualitative feedback from users consistently emphasized the need to visualize the virtual hands and suggested making minor adjustments to the sensitivity of soft-tissue deformation to improve realism. These insights will guide the next stages of development, ensuring the system is further optimized before real-world implementation.

## V. Conclusion

Difficult airway management requires extensive training and exposure to a broad spectrum of critical scenarios. Traditional training methods often fall short in replicating the cognitive and physical stress associated with complex real-world situations. To address these limitations, we developed and validated a VR-based simulation system that combines physical and virtual environments to deliver high-fidelity mixed-reality training capabilities. The customized sensing tools enable the collection of data on users’ procedural performance and biometric responses during simulations. Physics-based tissue deformation, calibrated and validated using experienced users, provides realistic interaction at the laryngoscope-airway interface, ensuring accurate synchronization between the physical and virtual realities.

The system demonstrates significant advantages over traditional simulation methods. Its ability to provide real-time haptic feedback, accurate soft-tissue deformation, and immersive visual environments addresses critical gaps in existing simulation technologies. However, limitations such as the lack of integrated evaluation tools for automated performance assessment remain. Compared to alternative systems, this approach will facilitate training and research in stress-inducing scenarios and simulating complex interactions, but further improvements are necessary to comprehensively assess its effectiveness.

Future work will focus on evaluating the system’s effectiveness in terms of user performance and skill development. While this study primarily relies on self-assessment by users to assess realism and accuracy, future iterations will incorporate objective metrics for quantifying training outcomes. This includes developing tools for automated performance evaluation and conducting comparative studies with traditional and alternative simulation modalities to establish the system’s broader efficacy and applicability.

In summary, this system represents a promising advancement in medical simulation. It lays the groundwork for future studies aimed at better evaluation methods and improving technical and cognitive performance in airway management.
